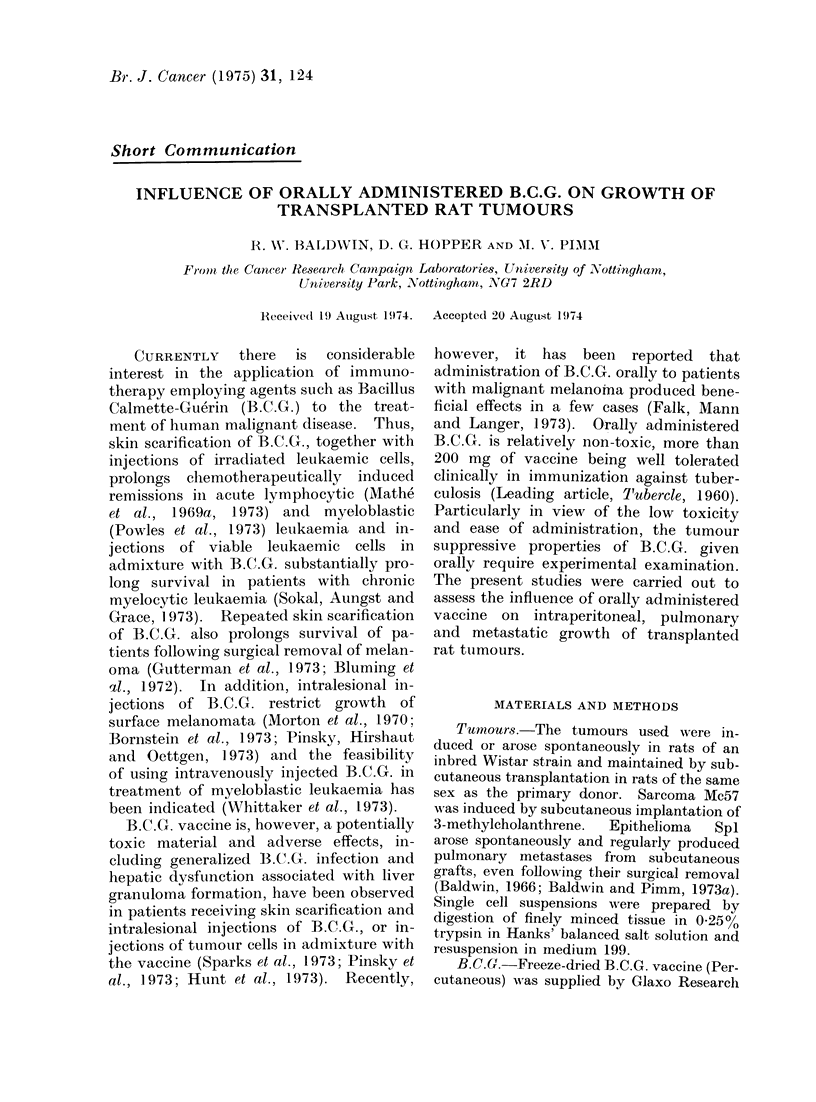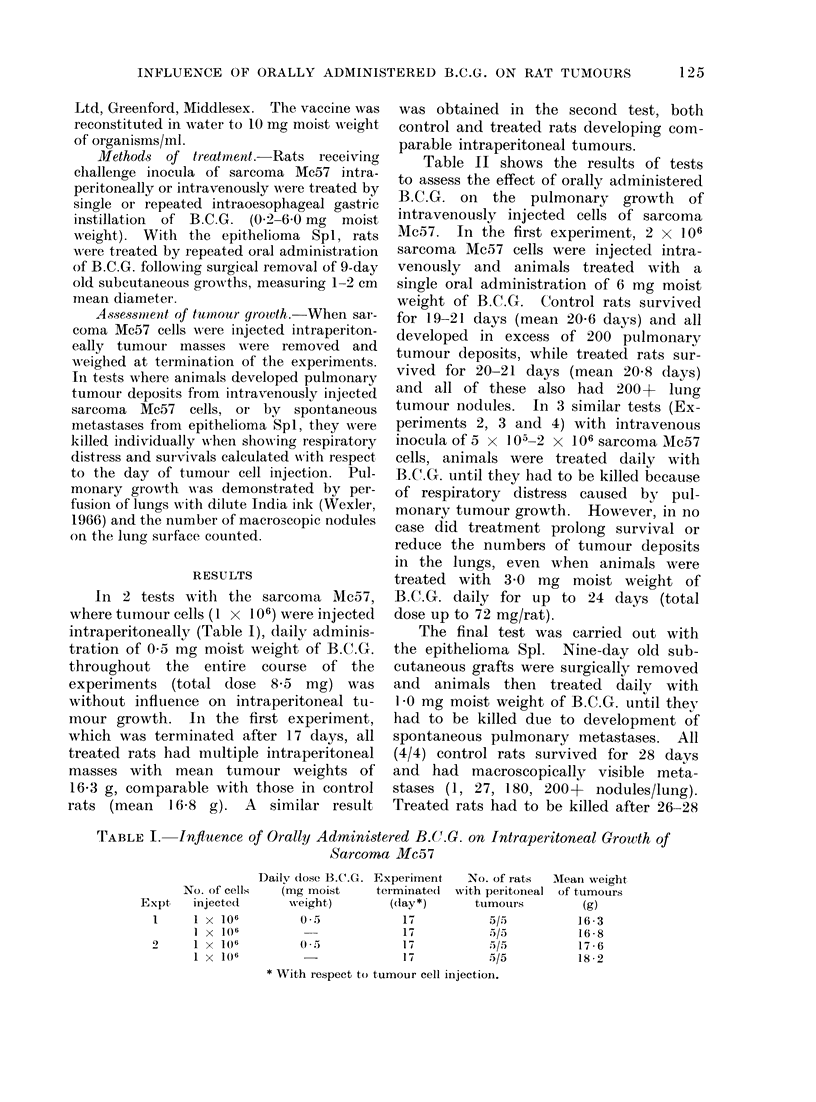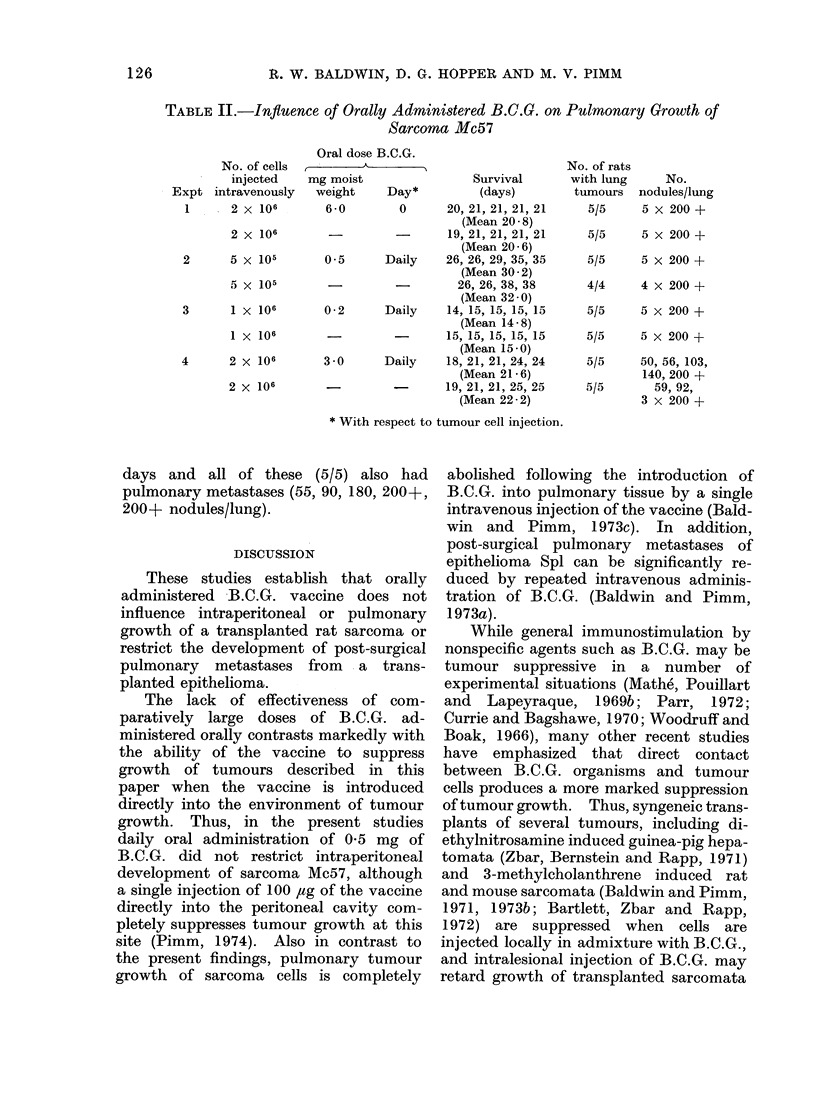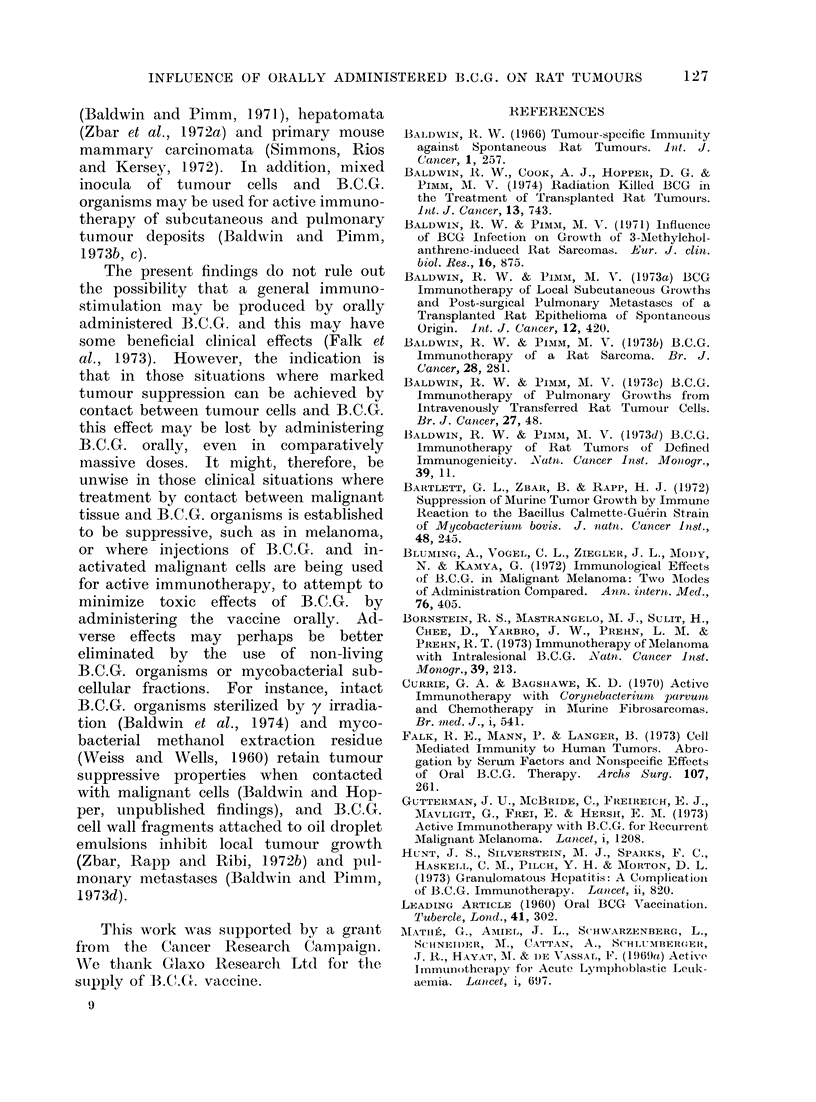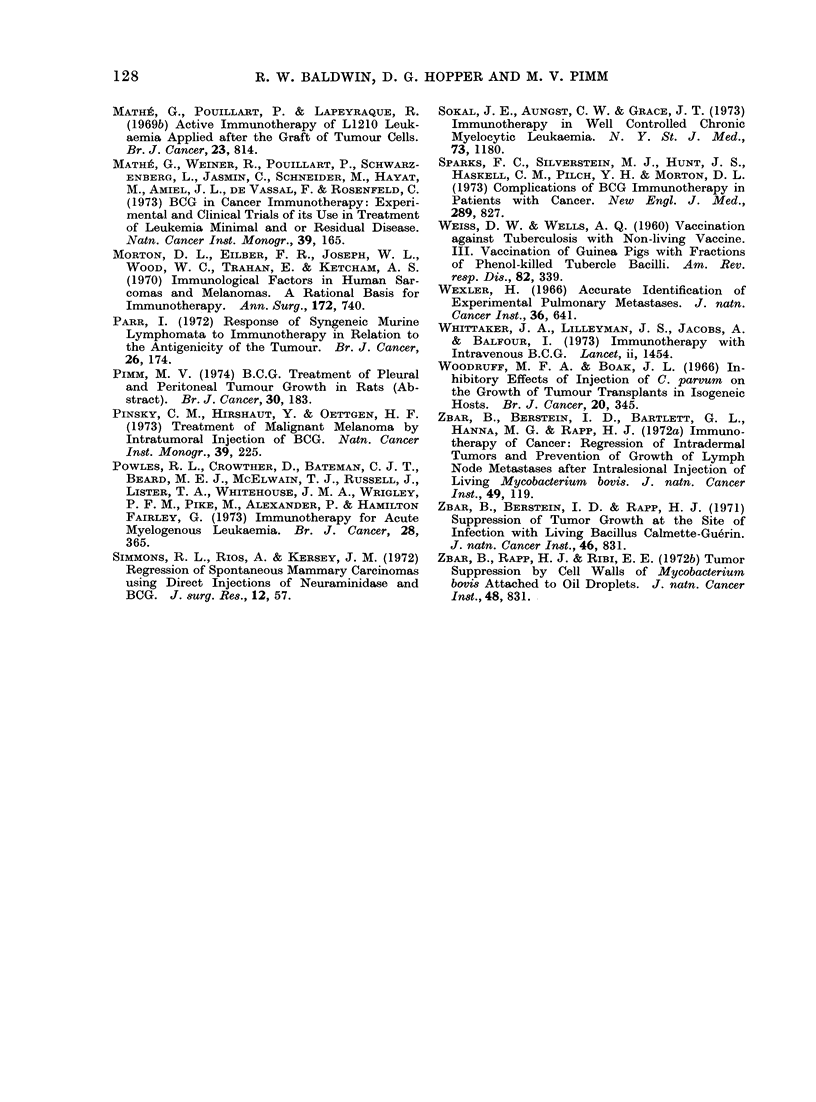# Influence of orally administered B.C.G. on growth of transplanted rat tumours.

**DOI:** 10.1038/bjc.1975.15

**Published:** 1975-01

**Authors:** R. W. Baldwin, D. G. Hopper, M. V. Pimm


*Br. J. Cancer* (1975) **31**, 124

***Short Communication***

### INFLUENCE OF ORALLY ADMINISTERED B.C.G. ON GROWTH OF TRANSPLANTED RAT TUMOURS

R. W. BALDWIN, D. G. HOPPER AND M. V. PIMM

*From the Cancer Research Campaign Laboratories, University of Nottingham, University Park, Nottingham, NG7 2RD*

Received 19 August 1974. Accepted 20 August 1974

CURRENTLY there is considerable interest in the application of immunotherapy employing agents such as Bacillus Calmette-Guérin (B.C.G.) to the treatment of human malignant disease. Thus, skin scarification of B.C.G., together with injections of irradiated leukaemic cells, prolongs chemotherapeutically induced remissions in acute lymphocytic (Mathé *et al.*, 1969*a*, 1973) and myeloblastic (Powles *et al.*, 1973) leukaemia and injections of viable leukaemic cells in admixture with B.C.G. substantially prolong survival in patients with chronic myelocytic leukaemia (Sokal, Aungst and Grace, 1973). Repeated skin scarification of B.C.G. also prolongs survival of patients following surgical removal of melanoma (Gutterman *et al.*, 1973; Bluming *et al.*, 1972). In addition, intralesional injections of B.C.G. restrict growth of surface melanomata (Morton *et al.*, 1970; Bornstein *et al.*, 1973; Pinsky, Hirshaut and Oettgen, 1973) and the feasibility of using intravenously injected B.C.G. in treatment of myeloblastic leukaemia has been indicated (Whittaker *et al.*, 1973).

B.C.G. vaccine is, however, a potentially toxic material and adverse effects, including generalized B.C.G. infection and hepatic dysfunction associated with liver granuloma formation, have been observed in patients receiving skin scarification and intralesional injections of B.C.G., or injections of tumour cells in admixture with the vaccine (Sparks *et al.*, 1973; Pinsky *et al.*, 1973; Hunt *et al.*, 1973). Recently, however, it has been reported that administration of B.C.G. orally to patients with malignant melanoma produced beneficial effects in a few cases (Falk, Mann and Langer, 1973). Orally administered B.C.G. is relatively non-toxic, more than 200 mg of vaccine being well tolerated clinically in immunization against tuberculosis (Leading article, *Tubercle*, 1960). Particularly in view of the low toxicity and ease of administration, the tumour suppressive properties of B.C.G. given orally require experimental examination. The present studies were carried out to assess the influence of orally administered vaccine on intraperitoneal, pulmonary and metastatic growth of transplanted rat tumours.

### MATERIALS AND METHODS

*Tumours.*—The tumours used were induced or arose spontaneously in rats of an inbred Wistar strain and maintained by subcutaneous transplantation in rats of the same sex as the primary donor. Sarcoma Mc57 was induced by subcutaneous implantation of 3-methylcholanthrene. Epithelioma Sp1 arose spontaneously and regularly produced pulmonary metastases from subcutaneous grafts, even following their surgical removal (Baldwin, 1966; Baldwin and Pimm, 1973*a*). Single cell suspensions were prepared by digestion of finely minced tissue in 0·25% trypsin in Hanks' balanced salt solution and resuspension in medium 199.

*B.C.G.*—Freeze-dried B.C.G. vaccine (Percutaneous) was supplied by Glaxo Research

Ltd, Greenford, Middlesex. The vaccine was reconstituted in water to 10 mg moist weight of organisms/ml.

*Methods of treatment.*—Rats receiving challenge inocula of sarcoma Mc57 intraperitoneally or intravenously were treated by single or repeated intraoesophageal gastric instillation of B.C.G. (0·2–6·0 mg moist weight). With the epithelioma Sp1, rats were treated by repeated oral administration of B.C.G. following surgical removal of 9-day old subcutaneous growths, measuring 1–2 cm mean diameter.

*Assessment of tumour growth.*—When sarcoma Mc57 cells were injected intraperitoneally tumour masses were removed and weighed at termination of the experiments. In tests where animals developed pulmonary tumour deposits from intravenously injected sarcoma Mc57 cells, or by spontaneous metastases from epithelioma Sp1, they were killed individually when showing respiratory distress and survivals calculated with respect to the day of tumour cell injection. Pulmonary growth was demonstrated by perfusion of lungs with dilute India ink (Wexler, 1966) and the number of macroscopic nodules on the lung surface counted.

### RESULTS

In 2 tests with the sarcoma Mc57, where tumour cells ($1 \times 10^6$) were injected intraperitoneally (Table I), daily administration of 0·5 mg moist weight of B.C.G. throughout the entire course of the experiments (total dose 8·5 mg) was without influence on intraperitoneal tumour growth. In the first experiment, which was terminated after 17 days, all treated rats had multiple intraperitoneal masses with mean tumour weights of 16·3 g, comparable with those in control rats (mean 16·8 g). A similar result was obtained in the second test, both control and treated rats developing comparable intraperitoneal tumours.

Table II shows the results of tests to assess the effect of orally administered B.C.G. on the pulmonary growth of intravenously injected cells of sarcoma Mc57. In the first experiment, $2 \times 10^6$ sarcoma Mc57 cells were injected intravenously and animals treated with a single oral administration of 6 mg moist weight of B.C.G. Control rats survived for 19–21 days (mean 20·6 days) and all developed in excess of 200 pulmonary tumour deposits, while treated rats survived for 20–21 days (mean 20·8 days) and all of these also had 200+ lung tumour nodules. In 3 similar tests (Experiments 2, 3 and 4) with intravenous inocula of $5 \times 10^5$–$2 \times 10^6$ sarcoma Mc57 cells, animals were treated daily with B.C.G. until they had to be killed because of respiratory distress caused by pulmonary tumour growth. However, in no case did treatment prolong survival or reduce the numbers of tumour deposits in the lungs, even when animals were treated with 3·0 mg moist weight of B.C.G. daily for up to 24 days (total dose up to 72 mg/rat).

The final test was carried out with the epithelioma Sp1. Nine-day old subcutaneous grafts were surgically removed and animals then treated daily with 1·0 mg moist weight of B.C.G. until they had to be killed due to development of spontaneous pulmonary metastases. All (4/4) control rats survived for 28 days and had macroscopically visible metastases (1, 27, 180, 200+ nodules/lung). Treated rats had to be killed after 26–28

TABLE I.—*Influence of Orally Administered B.C.G. on Intraperitoneal Growth of Sarcoma Mc57*

| Expt | No. of cells injected | Daily dose B.C.G. (mg moist weight) | Experiment terminated (day*) | No. of rats with peritoneal tumours | Mean weight of tumours (g) |
|---|---|---|---|---|---|
| 1 | $1 \times 10^6$ | 0·5 | 17 | 5/5 | 16·3 |
| | $1 \times 10^6$ | — | 17 | 5/5 | 16·8 |
| 2 | $1 \times 10^6$ | 0·5 | 17 | 5/5 | 17·6 |
| | $1 \times 10^6$ | — | 17 | 5/5 | 18·2 |

* With respect to tumour cell injection.

TABLE II.—*Influence of Orally Administered B.C.G. on Pulmonary Growth of Sarcoma Mc*57

| Expt | No. of cells injected intravenously | Oral dose B.C.G. mg moist weight | Oral dose B.C.G. Day* | Survival (days) | No. of rats with lung tumours | No. nodules/lung |
|---|---|---|---|---|---|---|
| 1 | $2 \times 10^6$ | 6·0 | 0 | 20, 21, 21, 21, 21 (Mean 20·8) | 5/5 | 5 × 200 + |
| | $2 \times 10^6$ | — | — | 19, 21, 21, 21, 21 (Mean 20·6) | 5/5 | 5 × 200 + |
| 2 | $5 \times 10^5$ | 0·5 | Daily | 26, 26, 29, 35, 35 (Mean 30·2) | 5/5 | 5 × 200 + |
| | $5 \times 10^5$ | — | — | 26, 26, 38, 38 (Mean 32·0) | 4/4 | 4 × 200 + |
| 3 | $1 \times 10^6$ | 0·2 | Daily | 14, 15, 15, 15, 15 (Mean 14·8) | 5/5 | 5 × 200 + |
| | $1 \times 10^6$ | — | — | 15, 15, 15, 15, 15 (Mean 15·0) | 5/5 | 5 × 200 + |
| 4 | $2 \times 10^6$ | 3·0 | Daily | 18, 21, 21, 24, 24 (Mean 21·6) | 5/5 | 50, 56, 103, 140, 200 + |
| | $2 \times 10^6$ | — | — | 19, 21, 21, 25, 25 (Mean 22·2) | 5/5 | 59, 92, 3 × 200 + |

* With respect to tumour cell injection.

days and all of these (5/5) also had pulmonary metastases (55, 90, 180, 200+, 200+ nodules/lung).

### DISCUSSION

These studies establish that orally administered B.C.G. vaccine does not influence intraperitoneal or pulmonary growth of a transplanted rat sarcoma or restrict the development of post-surgical pulmonary metastases from a transplanted epithelioma.

The lack of effectiveness of comparatively large doses of B.C.G. administered orally contrasts markedly with the ability of the vaccine to suppress growth of tumours described in this paper when the vaccine is introduced directly into the environment of tumour growth. Thus, in the present studies daily oral administration of 0·5 mg of B.C.G. did not restrict intraperitoneal development of sarcoma Mc57, although a single injection of 100 $\mu$g of the vaccine directly into the peritoneal cavity completely suppresses tumour growth at this site (Pimm, 1974). Also in contrast to the present findings, pulmonary tumour growth of sarcoma cells is completely abolished following the introduction of B.C.G. into pulmonary tissue by a single intravenous injection of the vaccine (Baldwin and Pimm, 1973*c*). In addition, post-surgical pulmonary metastases of epithelioma Spl can be significantly reduced by repeated intravenous administration of B.C.G. (Baldwin and Pimm, 1973*a*).

While general immunostimulation by nonspecific agents such as B.C.G. may be tumour suppressive in a number of experimental situations (Mathé, Pouillart and Lapeyraque, 1969*b*; Parr, 1972; Currie and Bagshawe, 1970; Woodruff and Boak, 1966), many other recent studies have emphasized that direct contact between B.C.G. organisms and tumour cells produces a more marked suppression of tumour growth. Thus, syngeneic transplants of several tumours, including diethylnitrosamine induced guinea-pig hepatomata (Zbar, Bernstein and Rapp, 1971) and 3-methylcholanthrene induced rat and mouse sarcomata (Baldwin and Pimm, 1971, 1973*b*; Bartlett, Zbar and Rapp, 1972) are suppressed when cells are injected locally in admixture with B.C.G., and intralesional injection of B.C.G. may retard growth of transplanted sarcomata

(Baldwin and Pimm, 1971), hepatomata (Zbar *et al.*, 1972*a*) and primary mouse mammary carcinomata (Simmons, Rios and Kersey, 1972). In addition, mixed inocula of tumour cells and B.C.G. organisms may be used for active immunotherapy of subcutaneous and pulmonary tumour deposits (Baldwin and Pimm, 1973*b*, *c*).

The present findings do not rule out the possibility that a general immunostimulation may be produced by orally administered B.C.G. and this may have some beneficial clinical effects (Falk *et al.*, 1973). However, the indication is that in those situations where marked tumour suppression can be achieved by contact between tumour cells and B.C.G. this effect may be lost by administering B.C.G. orally, even in comparatively massive doses. It might, therefore, be unwise in those clinical situations where treatment by contact between malignant tissue and B.C.G. organisms is established to be suppressive, such as in melanoma, or where injections of B.C.G. and inactivated malignant cells are being used for active immunotherapy, to attempt to minimize toxic effects of B.C.G. by administering the vaccine orally. Adverse effects may perhaps be better eliminated by the use of non-living B.C.G. organisms or mycobacterial subcellular fractions. For instance, intact B.C.G. organisms sterilized by $\gamma$ irradiation (Baldwin *et al.*, 1974) and mycobacterial methanol extraction residue (Weiss and Wells, 1960) retain tumour suppressive properties when contacted with malignant cells (Baldwin and Hopper, unpublished findings), and B.C.G. cell wall fragments attached to oil droplet emulsions inhibit local tumour growth (Zbar, Rapp and Ribi, 1972*b*) and pulmonary metastases (Baldwin and Pimm, 1973*d*).

This work was supported by a grant from the Cancer Research Campaign. We thank Glaxo Research Ltd for the supply of B.C.G. vaccine.

REFERENCES

Baldwin, R. W. (1966) Tumour-specific Immunity against Spontaneous Rat Tumours. *Int. J. Cancer*, **1**, 257.

Baldwin, R. W., Cook, A. J., Hopper, D. G. & Pimm, M. V. (1974) Radiation Killed BCG in the Treatment of Transplanted Rat Tumours. *Int. J. Cancer*, **13**, 743.

Baldwin, R. W. & Pimm, M. V. (1971) Influence of BCG Infection on Growth of 3-Methylcholanthrene-induced Rat Sarcomas. *Eur. J. clin. biol. Res.*, **16**, 875.

Baldwin, R. W. & Pimm, M. V. (1973*a*) BCG Immunotherapy of Local Subcutaneous Growths and Post-surgical Pulmonary Metastases of a Transplanted Rat Epithelioma of Spontaneous Origin. *Int. J. Cancer*, **12**, 420.

Baldwin, R. W. & Pimm, M. V. (1973*b*) B.C.G. Immunotherapy of a Rat Sarcoma. *Br. J. Cancer*, **28**, 281.

Baldwin, R. W. & Pimm, M. V. (1973*c*) B.C.G. Immunotherapy of Pulmonary Growths from Intravenously Transferred Rat Tumour Cells. *Br. J. Cancer*, **27**, 48.

Baldwin, R. W. & Pimm, M. V. (1973*d*) B.C.G. Immunotherapy of Rat Tumors of Defined Immunogenicity. *Natn. Cancer Inst. Monogr.*, **39**, 11.

Bartlett, G. L., Zbar, B. & Rapp, H. J. (1972) Suppression of Murine Tumor Growth by Immune Reaction to the Bacillus Calmette-Guérin Strain of *Mycobacterium bovis*. *J. natn. Cancer Inst.*, **48**, 245.

Bluming, A., Vogel, C. L., Ziegler, J. L., Mody, N. & Kamya, G. (1972) Immunological Effects of B.C.G. in Malignant Melanoma: Two Modes of Administration Compared. *Ann. intern. Med.*, **76**, 405.

Bornstein, R. S., Mastrangelo, M. J., Sulit, H., Chee, D., Yarbro, J. W., Prehn, L. M. & Prehn, R. T. (1973) Immunotherapy of Melanoma with Intralesional B.C.G. *Natn. Cancer Inst. Monogr.*, **39**, 213.

Currie, G. A. & Bagshawe, K. D. (1970) Active Immunotherapy with *Corynebacterium parvum* and Chemotherapy in Murine Fibrosarcomas. *Br. med. J.*, i, 541.

Falk, R. E., Mann, P. & Langer, B. (1973) Cell Mediated Immunity to Human Tumors. Abrogation by Serum Factors and Nonspecific Effects of Oral B.C.G. Therapy. *Archs Surg.* **107**, 261.

Gutterman, J. U., McBride, C., Freireich, E. J., Mavligit, G., Frei, E. & Hersh, E. M. (1973) Active Immunotherapy with B.C.G. for Recurrent Malignant Melanoma. *Lancet*, i, 1208.

Hunt, J. S., Silverstein, M. J., Sparks, F. C., Haskell, C. M., Pilch, Y. H. & Morton, D. L. (1973) Granulomatous Hepatitis: A Complication of B.C.G. Immunotherapy. *Lancet*, ii, 820.

Leading Article (1960) Oral BCG Vaccination. *Tubercle, Lond.*, **41**, 302.

Mathé, G., Amiel, J. L., Schwarzenberg, L., Schneider, M., Cattan, A., Schlumberger, J. R., Hayat, M. & de Vassal, F. (1969*a*) Active Immunotherapy for Acute Lymphoblastic Leukaemia. *Lancet*, i, 697.

MATHÉ, G., POUILLART, P. & LAPEYRAQUE, R. (1969b) Active Immunotherapy of L1210 Leukaemia Applied after the Graft of Tumour Cells. *Br. J. Cancer*, **23**, 814.

MATHÉ, G., WEINER, R., POUILLART, P., SCHWARZENBERG, L., JASMIN, C., SCHNEIDER, M., HAYAT, M., AMIEL, J. L., DE VASSAL, F. & ROSENFELD, C. (1973) BCG in Cancer Immunotherapy: Experimental and Clinical Trials of its Use in Treatment of Leukemia Minimal and or Residual Disease. *Natn. Cancer Inst. Monogr.*, **39**, 165.

MORTON, D. L., EILBER, F. R., JOSEPH, W. L., WOOD, W. C., TRAHAN, E. & KETCHAM, A. S. (1970) Immunological Factors in Human Sarcomas and Melanomas. A Rational Basis for Immunotherapy. *Ann. Surg.*, **172**, 740.

PARR, I. (1972) Response of Syngeneic Murine Lymphomata to Immunotherapy in Relation to the Antigenicity of the Tumour. *Br. J. Cancer*, **26**, 174.

PIMM, M. V. (1974) B.C.G. Treatment of Pleural and Peritoneal Tumour Growth in Rats (Abstract). *Br. J. Cancer*, **30**, 183.

PINSKY, C. M., HIRSHAUT, Y. & OETTGEN, H. F. (1973) Treatment of Malignant Melanoma by Intratumoral Injection of BCG. *Natn. Cancer Inst. Monogr.*, **39**, 225.

POWLES, R. L., CROWTHER, D., BATEMAN, C. J. T., BEARD, M. E. J., MCELWAIN, T. J., RUSSELL, J., LISTER, T. A., WHITEHOUSE, J. M. A., WRIGLEY, P. F. M., PIKE, M., ALEXANDER, P. & HAMILTON FAIRLEY, G. (1973) Immunotherapy for Acute Myelogenous Leukaemia. *Br. J. Cancer*, **28**, 365.

SIMMONS, R. L., RIOS, A. & KERSEY, J. M. (1972) Regression of Spontaneous Mammary Carcinomas using Direct Injections of Neuraminidase and BCG. *J. surg. Res.*, **12**, 57.

SOKAL, J. E., AUNGST, C. W. & GRACE, J. T. (1973) Immunotherapy in Well Controlled Chronic Myelocytic Leukaemia. *N. Y. St. J. Med.*, **73**, 1180.

SPARKS, F. C., SILVERSTEIN, M. J., HUNT, J. S., HASKELL, C. M., PILCH, Y. H. & MORTON, D. L. (1973) Complications of BCG Immunotherapy in Patients with Cancer. *New Engl. J. Med.*, **289**, 827.

WEISS, D. W. & WELLS, A. Q. (1960) Vaccination against Tuberculosis with Non-living Vaccine. III. Vaccination of Guinea Pigs with Fractions of Phenol-killed Tubercle Bacilli. *Am. Rev. resp. Dis.*, **82**, 339.

WEXLER, H. (1966) Accurate Identification of Experimental Pulmonary Metastases. *J. natn. Cancer Inst.*, **36**, 641.

WHITTAKER, J. A., LILLEYMAN, J. S., JACOBS, A. & BALFOUR, I. (1973) Immunotherapy with Intravenous B.C.G. *Lancet*, ii, 1454.

WOODRUFF, M. F. A. & BOAK, J. L. (1966) Inhibitory Effects of Injection of *C. parvum* on the Growth of Tumour Transplants in Isogeneic Hosts. *Br. J. Cancer*, **20**, 345.

ZBAR, B., BERSTEIN, I. D., BARTLETT, G. L., HANNA, M. G. & RAPP, H. J. (1972a) Immunotherapy of Cancer: Regression of Intradermal Tumors and Prevention of Growth of Lymph Node Metastases after Intralesional Injection of Living *Mycobacterium bovis*. *J. natn. Cancer Inst.*, **49**, 119.

ZBAR, B., BERSTEIN, I. D. & RAPP, H. J. (1971) Suppression of Tumor Growth at the Site of Infection with Living Bacillus Calmette-Guérin. *J. natn. Cancer Inst.*, **46**, 831.

ZBAR, B., RAPP, H. J. & RIBI, E. E. (1972b) Tumor Suppression by Cell Walls of *Mycobacterium bovis* Attached to Oil Droplets. *J. natn. Cancer Inst.*, **48**, 831.